# AMPK–PP1/PP2 axis regulates GSDMD-mediated pyroptosis via phosphorylation

**DOI:** 10.3389/fonc.2026.1737869

**Published:** 2026-08-18

**Authors:** Ting Zhang, Chunxiao Gao, Jixuan Xu, Weishan Zhang, Yamin Xing, Guangyuan Li, Mengxue Li, Yanwei Guo, Zisen Zhang, Pengyuan Zheng, Xiaoke Zheng, Xiufeng Chu

**Affiliations:** 1 Zhengzhou Key Laboratory of Lipid Metabolism and Precision Diagnosis and Treatment in Oncology, Oncology Department, The Fifth Affiliated Hospital of Zhengzhou University, Zhengzhou, China; 2 State Key Laboratory of Metabolic Dysregulation and Prevention and Treatment of Esophageal Cancer, Tianjian Laboratory of Advanced Biomedical Sciences, Zhengzhou, China; 3 Department of Gastrointestinal and Thyroid Surgery, The Fifth Affiliated Hospital of Zhengzhou University, Zhengzhou, China; 4 Marshall B. J. Medical Center, The Fifth Affiliated Hospital of Zhengzhou University, Zhengzhou, China; 5 Department of Oncology, The Second Affiliated Hospital, School of Medicine, The Chinese University of Hong Kong (Shenzhen), Longgang District People's Hospital of Shenzhen, Shenzhen, China; 6 Immunobiology and Transplant Science Center, Houston Methodist Hospital, Texas Medical Center, Houston, TX, United States

**Keywords:** AMPK, GSDMD, metformin, PP1/PP2, pyroptosis

## Abstract

Gasdermin D (GSDMD), the most important executor of pyroptosis, is activated through caspase cleavage to release the N-terminal fragment (GD-NT), which oligomerizes and forms pores in the plasma membrane to induce pyroptosis. We previously reported that AMP-activated protein kinase (AMPK) phosphorylates GD-NT, thereby inhibiting its cytolytic activity. Here, we further investigated the role of phosphorylation in GD-NT-mediated pyroptosis and found that the specific AMPK agonist A-769662 protected tumor cells from GD-NT-mediated pyroptosis and that metformin significantly reduced interleukin 1β (IL-1β) and IL-18 in the peritoneal fluid of lipopolysaccharide (LPS)-induced septic mice. Importantly, we identified PP1/PP2 as the key phosphatases that dephosphorylate GD-NT at Ser46. Treatment of cells with the phosphatase inhibitor okadaic acid (OA) significantly enhanced the phosphorylation of GD-NT and reduced GD-NT-mediated pyroptosis. Therefore, we conclude that the AMPK–PP1/PP2 axis is a regulatory mechanism in pyroptosis. Pharmacological targeting of AMPK–PP1/PP2 provides a potential strategy for pyroptosis-related diseases.

## Introduction

Pyroptosis is a lytic programmed cell death mediated by the gasdermin family. In response to pathogen infection or danger stimuli, caspases-1/-4/-5/-11 cleave GSDMD to release the cytolytic GD-NT. In turn, it oligomerizes and forms nanopores in the plasma membrane to cause osmotic imbalance and eventual cell rupture. The released inflammatory cellular contents can activate the innate immune response and cause excessive inflammation (1–3), thus making pyroptosis play important roles in human diseases, which include, but are not limited to, sepsis, autoimmune diseases, and cancer (4–6). Thus far, caspase-mediated cleavage of GSDMD activation has been well documented, such as inflammasome assembly and caspase-1 activation, intracellular lipopolysaccharide (LPS)-dependent activation of caspase-4/-5/-11, and ESCRT-III-mediated membrane repair (7). Nevertheless, how the biological activities of GD-NT, including its translocation, oligomerization, and pore-forming, are fine-tuned is poorly understood.

AMPK, the core regulator of cellular energy homeostasis, has been found to regulate cell survival by phosphorylating ULK1, RIPK1 (8, 9), and mTORC1 (10) and ferroptosis resistance through acetyl-CoA carboxylase (ACC) phosphorylation (11). In our previous work, we found that AMPK interacts with and phosphorylates GD-NT at Ser46 to inhibit its cytolytic activity (12). However, the applicability of this phosphorylation‑mediated regulation under other pathological conditions, as well as the identity of the eraser (dephosphorylating enzyme) responsible for this phosphorylation modification, has not been determined in that study. In the current study, we further investigated the regulatory axis of GD-NT phosphorylation in both tumor cells and macrophages. We found that pharmaceutical activation of AMPK suppressed pyroptosis in HeLa cells and Raw264.7 cells. In LPS-induced septic mice, metformin (Met) reduced the levels of IL-1β and IL18 in peritoneal fluid. In addition, we identified PP1 and PP2 as the phosphatases responsible for the removal of the phosphate group at GD-NT-Ser46. Thus, we reveal an AMPK–PP1/PP2 axis that regulates GSDMD-mediated pyroptosis.

## Results

### Phosphorylation at Ser46 inhibits GD-NT cytolytic activity in tumor cells

**Figure 1:**
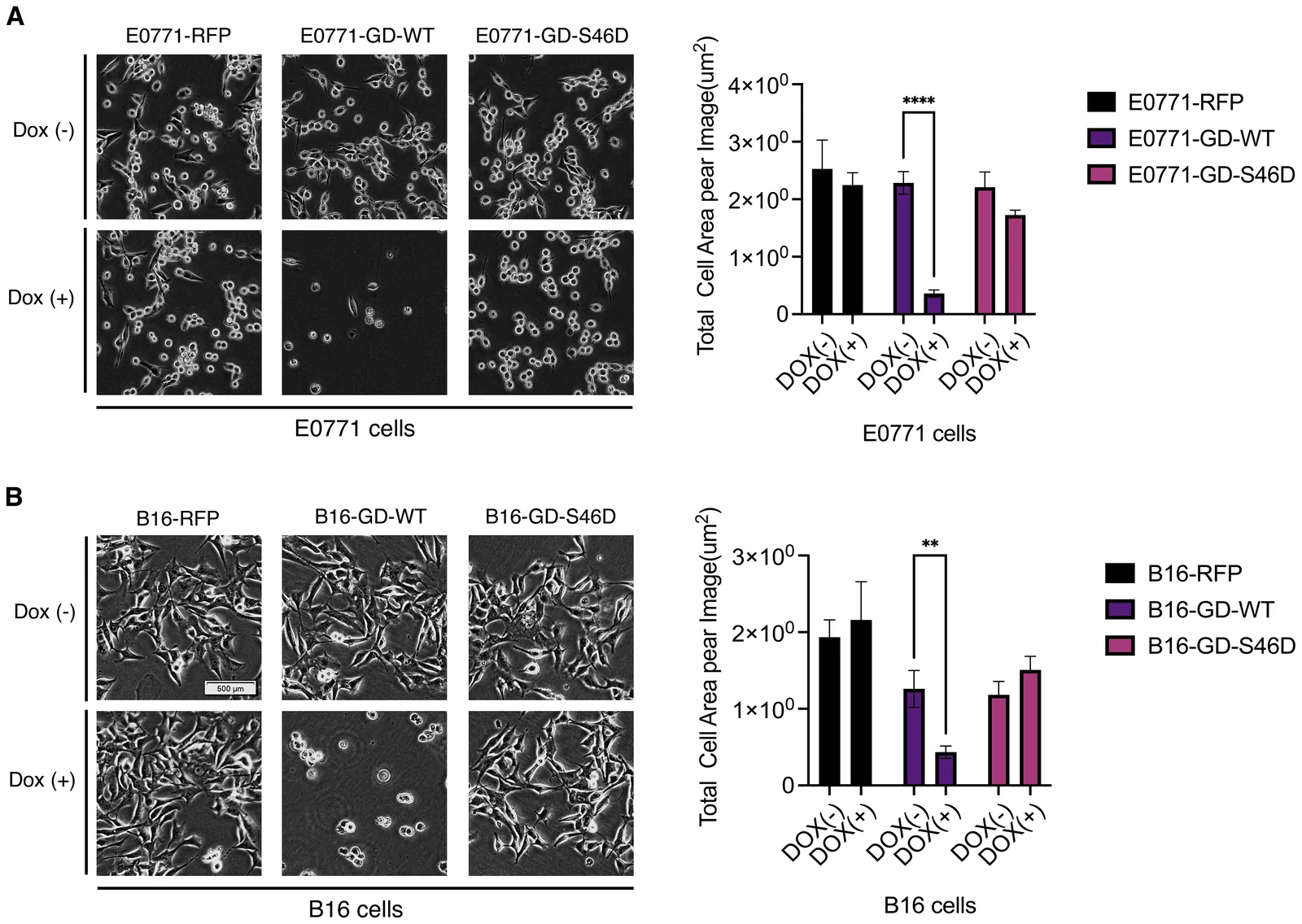
The effect of GD-NT-Ser46 phosphorylation on pyroptosis is verified in E0771 and B16 cells. **(A)** E0771 cells stably expressing Dox-inducible GD-NT-WT, GD-NT-S46D, or RFP control were treated with or without Dox. After 24 h, phase-contrast images were acquired. The total cell area in each image was quantified using ImageJ, and statistical analyses were performed using two-way ANOVA. **(B)** Similar to **(A)**, but for B16 cells. **P < 0.01 and ****P < 0.0001.

In our previous study, we found that AMPK activation suppresses pyroptosis by phosphorylating GD-NT at Ser46 in tumor cells. To further assess this effect, we used two additional tumor cell lines: the melanoma-derived B16 and the breast cancer-derived E0771. We transduced these tumor cells with the Tet-On lentiviral system to express GD-NT wild type (WT) or its constitutively phosphorylated mutant S46D in a doxycycline (Dox)-inducible manner. This design allows for a rigorous investigation into cytotoxic proteins and minimizes artificial effects between experimental groups. When treated with Dox, the cells expressing GD-NT-WT exhibited pyroptosis-specific morphological changes and a reduction of surviving cells. In contrast, survival status of the cells expressing GD-NT-S46D was barely affected by Dox treatment (**Figures 1A, B**). Consistently, the specific AMPK agonist A-769662 significantly inhibited pyroptosis in the cells that express Dox‑inducible GD‑NT‑WT (**Supplementary Figure 1A**). Thus, these data suggest that the inhibitory effect of AMPK on GD-NT-mediated pyroptosis occurs across a range of cell types.

### Metformin inhibits GSDMD-mediated pyroptosis in macrophages and septic mice

**Figure 2:**
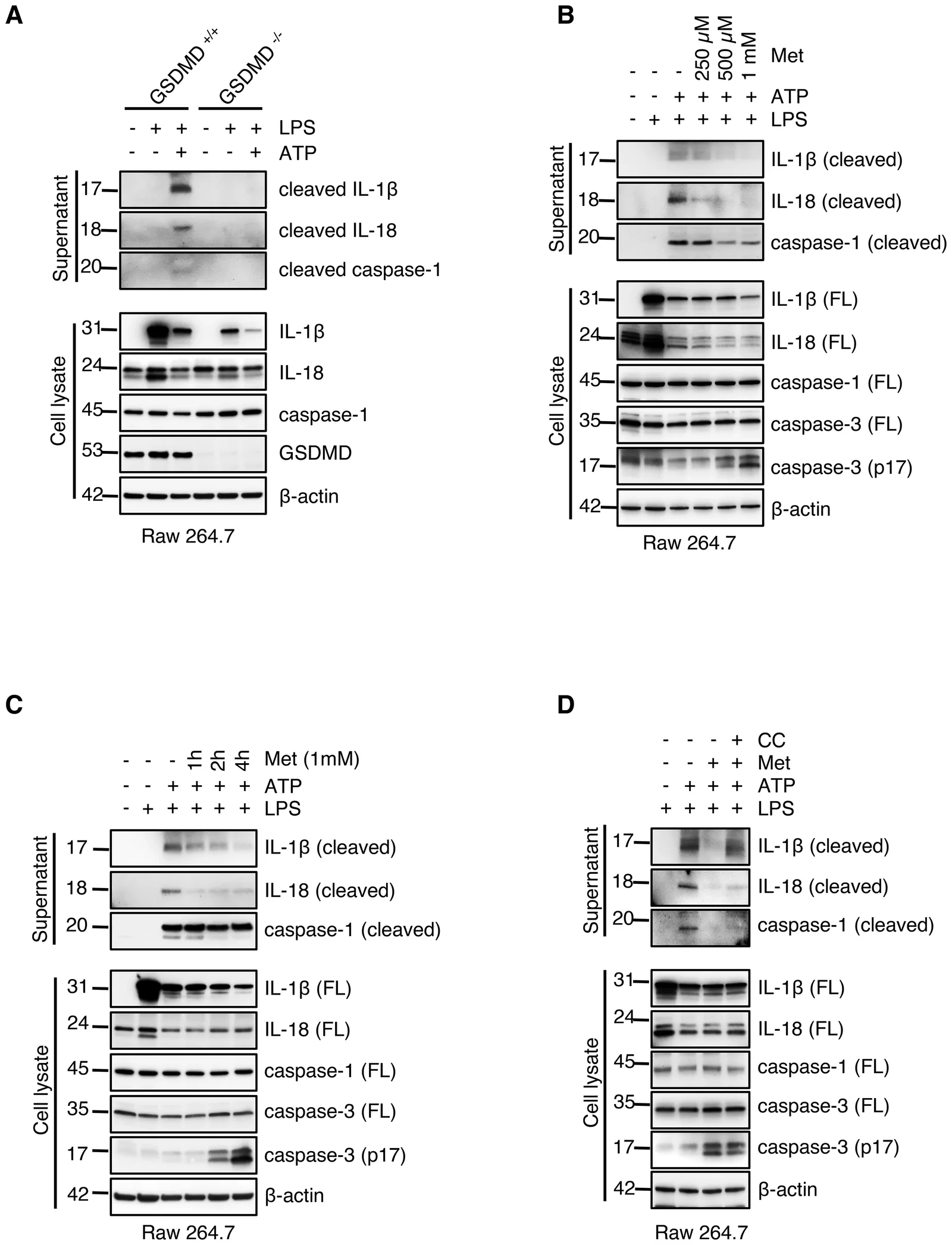
Pharmacological activation of AMPK attenuates GSDMD-mediated pyroptosis in macrophage. **(A)** Raw264.7 gasdermin D (GSDMD)-knockout cells were first treated with lipopolysaccharide (LPS; 500 ng/ml) for 4 h and then exposed to ATP (200 µM) for 4 h to induce pyroptosis. Metformin (Met, 1 mM) was added simultaneously with ATP. Proteins in the supernatant and whole cell lysate (WCL) were detected by Western blot. **(B)** Raw264.7 cells were treated with LPS (500 ng/ml) for 4 h and then exposed to ATP (200 µM) for 4 h to induce pyroptosis. Different concentrations of Met were added. Proteins in the supernatant and WCL were detected by Western blot. **(C)** Similar to **(B)**, but with Met added for 1, 2, or 4 h before LPS/ATP stimulation. Immunoblotting (IB) analysis was performed to analyze pyroptosis-related markers. **(D)** Raw264.7 cells were treated with LPS for 4 h and then simultaneously treated with ATP (200 µM) to induce pyroptosis. Met (1 mM) and compound C (10 µM) were added simultaneously with ATP for 4h. Proteins in the supernatant and WCL were detected by Western blot.

To assess the role of AMPK in GSDMD-mediated pyroptosis in macrophages, we used Raw264.7 cells and immortalized bone marrow-derived macrophages (iBMDMs). Firstly, we treated the cells with LPS/ATP to activate the classical NLRP3 inflammasome pathway. This led to caspase-1 cleavage/activation and the maturation of pro-inflammatory cytokines IL-1β and IL-18. These events were largely abolished by genetic knockout of GSDMD, verifying the occurrence of GSDMD-mediated pyroptosis in Raw264.7 cells (**Figure 2A**). Subsequently, the AMPK agonist Met and A-769662 were used to enhance AMPK activity. We found that the AMPK agonists Met and A-769662 significantly suppressed pyroptosis in RAW 264.7 cells and iBMDMs, respectively (**Figures 2B, C**; **Supplementary Figure 1B**), the AMPK antagonist compound C (CC) counteracted the protective effect of Met in pyroptosis (**Figure 2D**).

**Figure 3:**
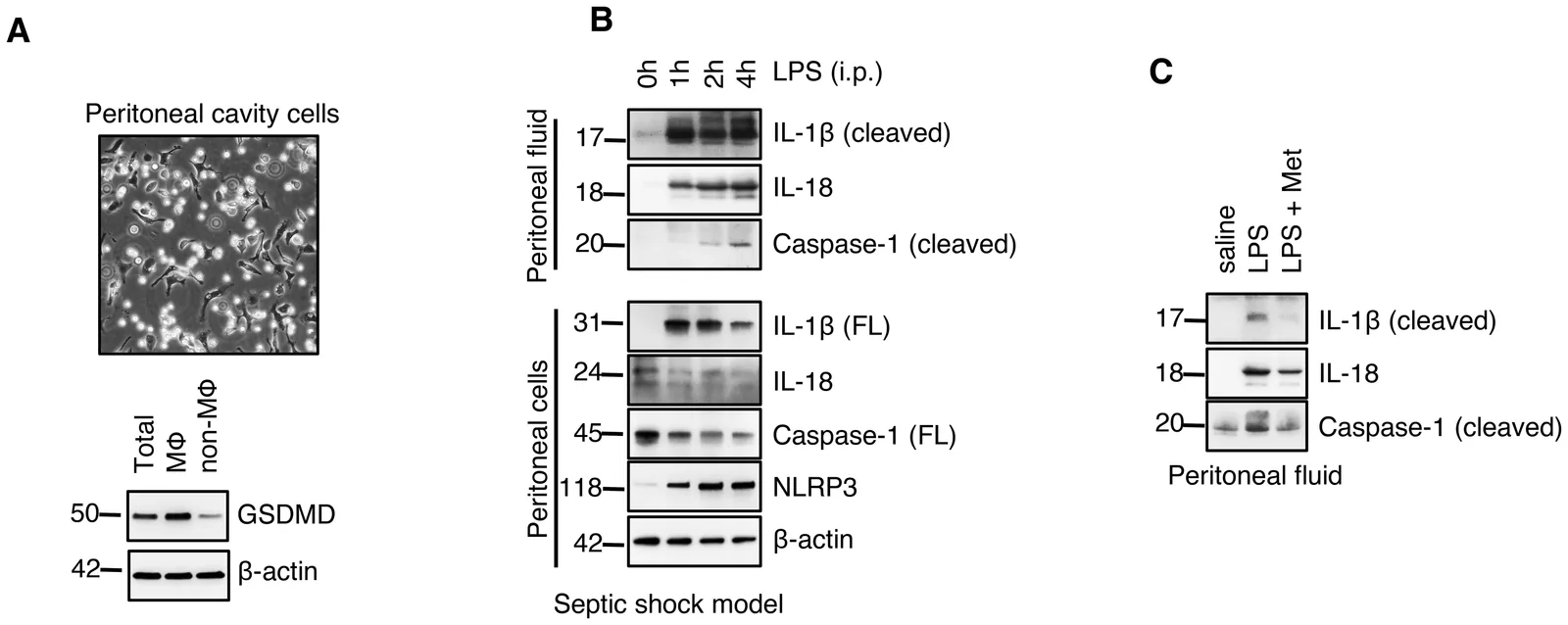
Metformin attenuates GSDMD-mediated pyroptosis in septic mice. **(A)** Peritoneal cavity cells were collected and cultured *in vitro* for 4 h. Floating cells were regarded as macrophages (*Mφ*), while non-floating cells were considered as non-macrophages (*non-Mφ*). GSDMD in total macrophages (*Total Mφ*), Mφ, and non-Mφ was analyzed by Western blotting. Representative phase-contrast images of peritoneal cells are also shown. **(B)** Lipopolysaccharide (LPS; 20 mg/kg) was injected intraperitoneally to induce endotoxic shock. Peritoneal fluid and peritoneal cells were collected at 0, 1, 2, and 4 h after LPS injection, and the proteins were extracted and analyzed by immunoblotting to evaluate changes in the pyroptosis-related proteins. **(C)** Mice were given intraperitoneal injections of saline, LPS alone, or LPS (20 mg/kg) combined with Met (100mg/kg) for 4 h. The protein levels in the peritoneal fluid of mice were then measured. Data are representative of at least two independent experiments.

Based on our *in vitro* finding that Met blocks GD-NT-mediated pyroptosis, we further evaluated this effect in LPS-induced septic mice. We found that, among the cells residing in the peritoneal cavity, macrophages are the predominant population expressing GSDMD (**Figures 3A**). Moreover, increased levels of IL‑1β and IL‑18 in the peritoneal lavage fluid were observed over time following intraperitoneal injection of LPS, and treating mice with Met significantly reduced the release of IL-1β and IL-18 in the peritoneal lavage fluid (**Figures 3B, C**). Although these in vivo findings are preliminary, they nevertheless suggest that AMPK regulates GSDMD-mediated pyroptosis not just in cancer cells but also in macrophages.

### Phosphorylation at GD-NT-Ser46 is enhanced by okadaic acid

**Figure 4:**
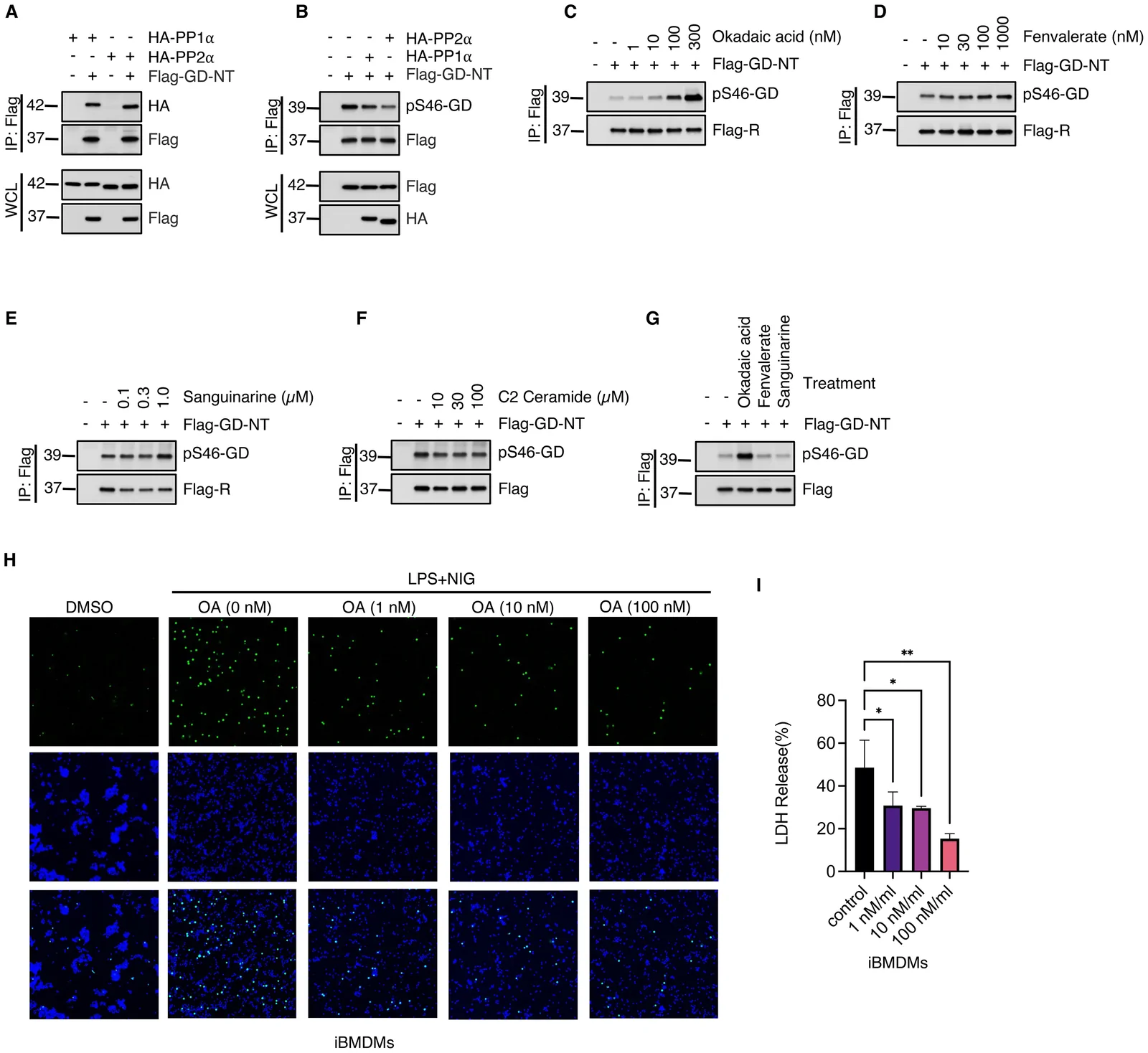
Phosphatase inhibition enhances phosphorylation at GD-NT-Ser46. **(A)** Interaction of protein phosphatase and gasdermin D (GSDMD). IB analysis of anti-Flag immunoprecipitation (IP) and WCL derived from HEK293 cells co-transfected with Flag-GSDMD and/or HA-PP1α or HA-PP2α. **(B)** Similar to **(A)**, except that the purpose here was to assess the influence of PP1α or PP2α on GSDMD phosphorylation. Data are representative of at least two independent experiments. **(C–F)** HEK293 cells were transfected with Flag-GD-NT. After 16 h, cells were harvested for anti-Flag IP assay and IB analysis with indicated antibodies. Before harvesting, cells were treated with okadaic acid (OA), fenvalerate, sanguinarine, C2 ceramide, or dimethyl sulfoxide (DMSO) for 2 h. Data are representative of at least two independent experiments. **(G)** HEK293 cells were transfected with Flag-GD-NT, followed by anti-Flag IP assay and IB analysis with indicated antibodies. Cells were treated with OA (0.3 μM), fenvalerate (1 μM), sanguinarine (1 μM), or DMSO for 2 h before harvest. **(H)** iBMDM cells were treated with LPS (1 µg/ml, 6 h) followed by nigericin (2 µM, 3 h). Different concentrations of OA were added at the same time as nigericin. The cells were stained with Hoechst and SYTOX Green and subjected to phase-contrast imaging and fluorescence microscopy. SYTOX-positive cells were considered dead cells. **(I)** Similar to **(H)**, except with the determination of the lactate dehydrogenase (LDH) level. *P < 0.05 and **P < 0.01.

Phosphorylation is a dynamic and reversible post-translational modification regulated by kinases and phosphatases. Among the protein phosphatases, PP1α and PP2α have been reported to dephosphorylate diverse substrates (13–16). To investigate whether PP1α and PP2α regulate the phosphorylation at GD-NT-Ser46, we examined their association with GD-NT by co-immunoprecipitation (Co-IP). It was found that PP1α and PP2α interacted with GD-NT (**Figure 4A**). Furthermore, PP1α and PP2α reduced the phosphorylation at GD-NT-Ser46 (**Figure 4B**). Subsequently, we examined the effects of phosphatase modulators, including okadaic acid (OA), fenvalerate, sanguinarine, and ceramide (**Figures 4C–F**), on GD-NT phosphorylation. The results showed that OA markedly enhanced phosphorylation at GD-NT-Ser46 (**Figures 4C–G**). To assess the impact of OA on GSDMD-mediated pyroptosis, iBMDMs were employed. The morphological analysis and lactate dehydrogenase (LDH) release assays revealed that OA significantly attenuated LPS/nigericin-induced macrophage pyroptosis (**Figures 4H, I**). These results support the important role of PP1/PP2 in regulating GSDMD phosphorylation and its pyroptotic activity.

## Discussion

Protein phosphorylation is a key mechanism that regulates inflammasome activation and pyroptosis at multiple steps (17–19). For instance, protein kinase A (PKA) phosphorylates NLRP3 at Ser295 to reduce NLRP3 oligomerization and limit inflammasome assembly, leading to a lower baseline IL-1β secretion (20–23). Conversely, Pyk2-dependent phosphorylation of the adaptor ASC (apoptosis-associated Speck-like protein containing a CARD) at Tyr146 promotes ASC oligomerization and enhances caspase-1 activation (24, 25). Beyond these upstream checkpoints, AMPK links cellular energy to inflammatory signaling and affects pyroptosis through several reported pathways. For example, AMPK phosphorylates RIPK1 at Ser321, thereby suppressing the RIPK1–caspase-8–GSDMD axis (26). In addition, AMPK phosphorylates gasdermin E (GSDME) at Thr6 to reduce caspase-3-mediated GSDME cleavage (27, 28). Our previous work demonstrated that the AMPK-mediated phosphorylation of GD-NT at Ser46 restricts GD-NT membrane targeting and oligomerization, thereby weakening pore formation in tumor models (12).

In the present study, we further defined the phosphorylation at the same residue of GSDMD. We found that AMPK has a protective role in GSDMD-mediated pyroptosis not just in tumor cells but also in macrophages, as well as in septic mice. A more important finding is that PP1/PP2 interacts with and dephosphorylates GD-NT, thereby restoring pyroptotic activity. Interestingly, Li et al. (29) reported that PP1 dephosphorylates GSDMD at Thr213, implying that PP1/PP2 regulates GSDMD not just at one single site but at multiple distinct sites.

Beyond our findings, several open questions remain. Firstly, our work focuses on the role of AMPK in the post-cleavage regulation of GSDMD, we did not assess its effects on upstream events such as NLRP3 oligomerization and ASC speck formation. Therefore, we cannot exclude the involvement of additional mechanisms in the observed phenotypes in septic mice. Secondly, the potential spatiotemporal crosstalk among distinct phosphorylation sites of GD-NT, such as Ser46 and Thr213, remains unclear. Future work is needed to integrate multi-site phosphoproteomic profiling to systematically delineate the hierarchical regulatory logic governing GD-NT cytolytic activity. Thirdly, although the use of OA helps in the assessment of the contribution of PP1/PP2 to the regulation of GD-NT phosphorylation, its poor selectivity and dose-dependent cytotoxicity limit its potential clinical use. The development of novel chemical agents that specifically target the AMPK–PP1/PP2–GSDMD interaction interface is a key focus for future research.

## Methods

### Data reporting

No statistical methods were used to predetermine the sample size. The experiments were not randomized. Investigators were not blinded to allocation during the experiments and outcome assessment.

### Cell culture and treatment

The E0771, B16, HEK293, and iBMDM cells were purchased from ATCC (Manassas, VA, USA). Raw264.7 cells were gifted by Daniel A. Bachovchin. The cells were cultured in Dulbecco’s modified Eagle’s medium (DMEM) supplemented with 10% heat-inactivated fetal bovine serum (FBS), 100 U/ml penicillin, and 100 µg/ml streptomycin. All cells were cultured at 37°C in a 5% CO_2_ incubator. All cells were tested for mycoplasma contamination using PCR and morphologically identified. Raw264.7 cells were stimulated with LPS (500 ng/mL) for 4 h, followed by ATP (200 µM) for 4 h to activate the canonical NLRP3 inflammasome pathway.

### Co-immunoprecipitation with Western blot

For the lysis buffer, erythrocyte blood cell (EBC) buffer (50 mM Tris, pH 7.5, 120 mM NaCl, 0.5% NP-40) containing protease/phosphatase inhibitors was used.

The antibodies used were: AMPKα1, AMPKα2, p-AMPK (Thr172) (#2795/#2535; Cell Signaling Technology, Danvers, MA, USA); Flag (F1804; Sigma, St. Louis, MO, USA), HA (H6908; Sigma, St. Louis, MO, USA), GSDMD (ab219800; Abcam, Cambridge, UK), and custom p-GSDMD (Ser46) antibody (Abclonal, Woburn, MA, USA).

### Construction of plasmids and stable cell lines

Plasmids were obtained from pDB-His-MBP-mGSDMD (123365) and pECE-HA-AMPKα1/α2 (69504/31654; both from Addgene, Watertown, MA, USA). The CRISPR-Cas9 vector was pX462-hPRKAA1/2-gRNA (74374-74377; Addgene, Watertown, MA, USA).

For mutant construction, the GSDMD-S46A/D/E mutant and the truncated mutant (Δ39-53) were generated using the QuickChange site-directed mutagenesis kit (Agilent, Santa Clara, CA, USA).

For stable cell lines, E0771, B16, and other cells were transduced with a lentivirus packaging system (pSPAX2/pMD2.G) and selected by puromycin (2 μg/ml).

### Protein enrichment from the culture medium or peritoneal fluid

To enrich the proteins in the culture medium, 1 ml of the culture medium was centrifuged at 14,000 × *g* for 10 min at 4°C to remove cellular debris. Afterward, 10 µl StrataClean resin (400714; Agilent, Santa Clara, CA, USA) was added for 1 h incubation on a rotator at 4°C. The supernatant was removed by centrifugation. The resin was harvested and suspended in 50 µl 2× loading buffer for immunoblotting.

In septic mice, the proteins in flushed peritoneal fluid were also enriched in the same manner.

### Cytotoxicity assay and cell viability assay

Cell death and cell viability were assessed using the Non-Radioactive Cytotoxicity Assay Kit (G1780) and the CellTiter-Glo Luminescent Cell Viability Assay Kit (G7571; both from Promega, Madison, WI, USA), respectively. Briefly, 5 × 10^3^ cells were cultured in 96-well plates with an opaque wall. At the desired time points, cell death was determined by titrating the amount of LDH released into the culture medium, while cell viability was determined based on the ATP levels within cells according to the manufacturer’s instructions.

### Hoechst and SYTOX™ Green staining

The Hoechst staining solution was prepared by diluting the Hoechst stock solution at 1:2,000 in phosphate-buffered saline (PBS). The SYTOX Green staining solution was prepared by diluting the stock solution at a ratio of 1:30,000 (167 nM) in phosphate-free buffer. For cell staining, the medium was then removed and sufficient staining solution was added to cover the cells. After incubation, the supernatant was discarded, and the cells were rinsed three times prior to microscopic observation.

### Animal models

The mouse strains were WT C57BL/6, female mice. For model establishment, in the LPS-induced intraperitoneal pyroptosis model, LPS (20 mg/kg) was intraperitoneally injected and the peritoneal lavage fluid collected 4 h later. The levels of IL-1β and IL-18 were then determined. Met was administered via intraperitoneal (i.p.) injection at a dose of 100 mg/kg for 30 min before LPS challenge.

### Statistical analysis

Inter-group differences were analyzed using Inter-group differences were analyzed using one-way or two-way ANOVA, as appropriate, followed by Bonferroni’s *post-hoc* multiple-comparisons test (for multigroup comparisons) in GraphPad Prism, version 10 (GraphPad Software, San Diego, CA, USA). Statistical significance was defined as *p* < 0.05.

## Data Availability

The original contributions presented in the study are included in the article/**Supplementary Material**. Further inquiries can be directed to the corresponding authors.

## References

[1] Liu X Zhang Z Ruan J Pan Y Magupalli VG Wu H . Inflammasome-activated gasdermin D causes pyroptosis by forming membrane pores. Nature. (2016) 535:153–8. doi: 10.1038/nature18629 27383986 PMC5539988

[2] Shi J Zhao Y Wang K Shi X Wang Y Huang H . Cleavage of GSDMD by inflammatory caspases determines pyroptotic cell death. Nature. (2015) 526:660–5. doi: 10.1038/nature15514 26375003

[3] Kayagaki N Stowe IB Lee BL O’Rourke K Anderson K Warming S . Caspase-11 cleaves gasdermin D for non-canonical inflammasome signalling. Nature. (2015) 526:666–71. doi: 10.1038/nature15541 26375259

[4] Rathkey JK Zhao J Liu Z Chen Y Yang J Kondolf HC . Chemical disruption of the pyroptotic pore-forming protein gasdermin D inhibits inflammatory cell death and sepsis. Sci Immunol. (2018) 3(26):eaat2738. doi: 10.1126/sciimmunol.aat2738 30143556 PMC6462819

[5] Zhang D Li Y Du C Sang L Liu L Li Y . Evidence of pyroptosis and ferroptosis extensively involved in autoimmune diseases at the single-cell transcriptome level. J Transl Med. (2022) 20(1):363. doi: 10.1186/s12967-022-03566-6 35962439 PMC9373312

[6] Zhang Z Zhang Y Xia S Kong Q Li S Liu X . Gasdermin E suppresses tumour growth by activating anti-tumour immunity. Nature. (2020) 579:415–20. doi: 10.1038/s41586-020-2071-9 32188940 PMC7123794

[7] Rühl S Shkarina K Demarco B Heilig R Santos JC Broz P . ESCRT-dependent membrane repair negatively regulates pyroptosis downstream of GSDMD activation. Science. (2018) 362:956–60. doi: 10.1126/science.aar7607 30467171

[8] Kim J Kundu M Viollet B Guan K-L . AMPK and mTOR regulate autophagy through direct phosphorylation of Ulk1. Nat Cell Biol. (2011) 13:132–41. doi: 10.1038/ncb2152 21258367 PMC3987946

[9] Hardie DG Ross FA Hawley SA . AMPK: A nutrient and energy sensor that maintains energy homeostasis. Nat Rev Mol Cell Biol. (2012) 13:251–62. doi: 10.1038/nrm3311 22436748 PMC5726489

[10] Inoki K Zhu T Guan K-L . TSC2 mediates cellular energy response to control cell growth and survival. Cell. (2003) 115:577–90. doi: 10.1016/s0092-8674(03)00929-2 14651849

[11] Lee H Zandkarimi F Zhang Y Meena JK Kim J Zhuang L . Energy-stress-mediated AMPK activation inhibits ferroptosis. Nat Cell Biol. (2020) 22:225–34. doi: 10.1038/s41556-020-0461-8 32029897 PMC7008777

[12] Chu X Xiao X Wang G Uosef A Lou X Arnold P . Gasdermin D-mediated pyroptosis is regulated by AMPK-mediated phosphorylation in tumor cells. Cell Death Dis. (2023) 14(7):469. doi: 10.1038/s41419-023-06013-6 37495617 PMC10372026

[13] Zhou Y-R Dang J-J Yang Q-C Sun Z-J . The regulation of pyroptosis by post-translational modifications: Molecular mechanisms and therapeutic targets. eBioMedicine. (2024) 109:105420. doi: 10.1016/j.ebiom.2024.105420 39476537 PMC11564932

[14] Hoermann B Kokot T Helm D Heinzlmeir S Chojnacki JE Schubert T . Dissecting the sequence determinants for dephosphorylation by the catalytic subunits of phosphatases PP1 and PP2A. Nat Commun. (2020) 11(1):3583. doi: 10.1038/s41467-020-17334-x 32681005 PMC7367873

[15] Ning J Sala M Reina J Kalagiri R Hunter T McCullough BS . Histidine phosphorylation: Protein kinases and phosphatases. IJMS. (2024) 25:7975. doi: 10.3390/ijms25147975 39063217 PMC11277029

[16] Bertolotti A . The split protein phosphatase system. Biochem J. (2018) 475:3707–23. doi: 10.1042/bcj20170726 30523060 PMC6282683

[17] O’Keefe ME Dubyak GR Abbott DW . Post-translational control of NLRP3 inflammasome signaling. J Biol Chem. (2024) 300:107386. doi: 10.1016/j.jbc.2024.107386 38763335 PMC11245928

[18] Xu W Huang Y . Regulation of inflammatory cell death by phosphorylation. Front Immunol. (2022) 13:851169. doi: 10.3389/fimmu.2022.851169 35300338 PMC8921259

[19] McKee CM Fischer FA Bezbradica JS Coll RC . PHOrming the inflammasome: Phosphorylation is a critical switch in inflammasome signalling. Biochem Soc Trans. (2021) 49:2495–507. doi: 10.1042/BST20200987 34854899 PMC8786285

[20] Zhong B Sun S Tan KS Ong HH Du J Liu F . Hypoxia-inducible factor 1α activates the NLRP3 inflammasome to regulate epithelial differentiation in chronic rhinosinusitis. J Allergy Clin Immunol. (2023) 152:1444–1459.e14. doi: 10.1016/j.jaci.2023.09.020 37777019

[21] Zhao W Shi C-S Harrison K Hwang I-Y Nabar NR Wang M . AKT regulates NLRP3 inflammasome activation by phosphorylating NLRP3 serine 5. J Immunol. (2020) 205:2255–64. doi: 10.4049/jimmunol.2000649 32929041 PMC7541779

[22] Mortimer L Moreau F MacDonald JA Chadee K . NLRP3 inflammasome inhibition is disrupted in a group of auto-inflammatory disease CAPS mutations. Nat Immunol. (2016) 17:1176–86. doi: 10.1038/ni.3538 27548431

[23] Song N Li T . Regulation of NLRP3 inflammasome by phosphorylation. Front Immunol. (2018) 9:2305. doi: 10.3389/fimmu.2018.02305 30349539 PMC6186804

[24] Mambwe B Neo K Javanmard Khameneh H Leong KWK Colantuoni M Vacca M . Tyrosine dephosphorylation of ASC modulates the activation of the NLRP3 and AIM2 inflammasomes. Front Immunol. (2019) 10:1556. doi: 10.3389/fimmu.2019.01556 31333677 PMC6624653

[25] Liang Z Damianou A Di Daniel E Kessler BM . Inflammasome activation controlled by the interplay between post-translational modifications: Emerging drug target opportunities. Cell Commun Signal. (2021) 19(1):23. doi: 10.1186/s12964-020-00688-6 33627128 PMC7905589

[26] Yang Y Fang H Xie Z Ren F Yan L Zhang M . Yersinia infection induces glucose depletion and AMPK-dependent inhibition of pyroptosis in mice. Nat Microbiol. (2024) 9:2144–59. doi: 10.1038/s41564-024-01734-6 38844594

[27] Ai Y Wang W Liu F Fang W Chen H Wu L . Mannose antagonizes GSDME-mediated pyroptosis through AMPK activated by metabolite GlcNAc-6P. Cell Res. (2023) 33:904–22. doi: 10.1038/s41422-023-00848-6 37460805 PMC10709431

[28] Devant P Kagan JC . Molecular mechanisms of gasdermin D pore-forming activity. Nat Immunol. (2023) 24:1064–75. doi: 10.1038/s41590-023-01526-w 37277654 PMC12379974

[29] Li Y Pu D Huang J Zhang Y Yin H . Protein phosphatase 1 regulates phosphorylation of gasdermin D and pyroptosis. Chem Commun. (2022) 58:11965–8. doi: 10.1039/d2cc03590a 36205355

