# Supplementary material for: AMPK–PP1/PP2 axis regulates GSDMD-mediated pyroptosis via phosphorylation
Source: Front Oncol. 2026 Aug 18;16:1737869. doi: 10.3389/fonc.2026.1737869 (PMC13529552; doi:10.3389/fonc.2026.1737869)
Supplement: Supplementary file 1 [file Image1.pdf]

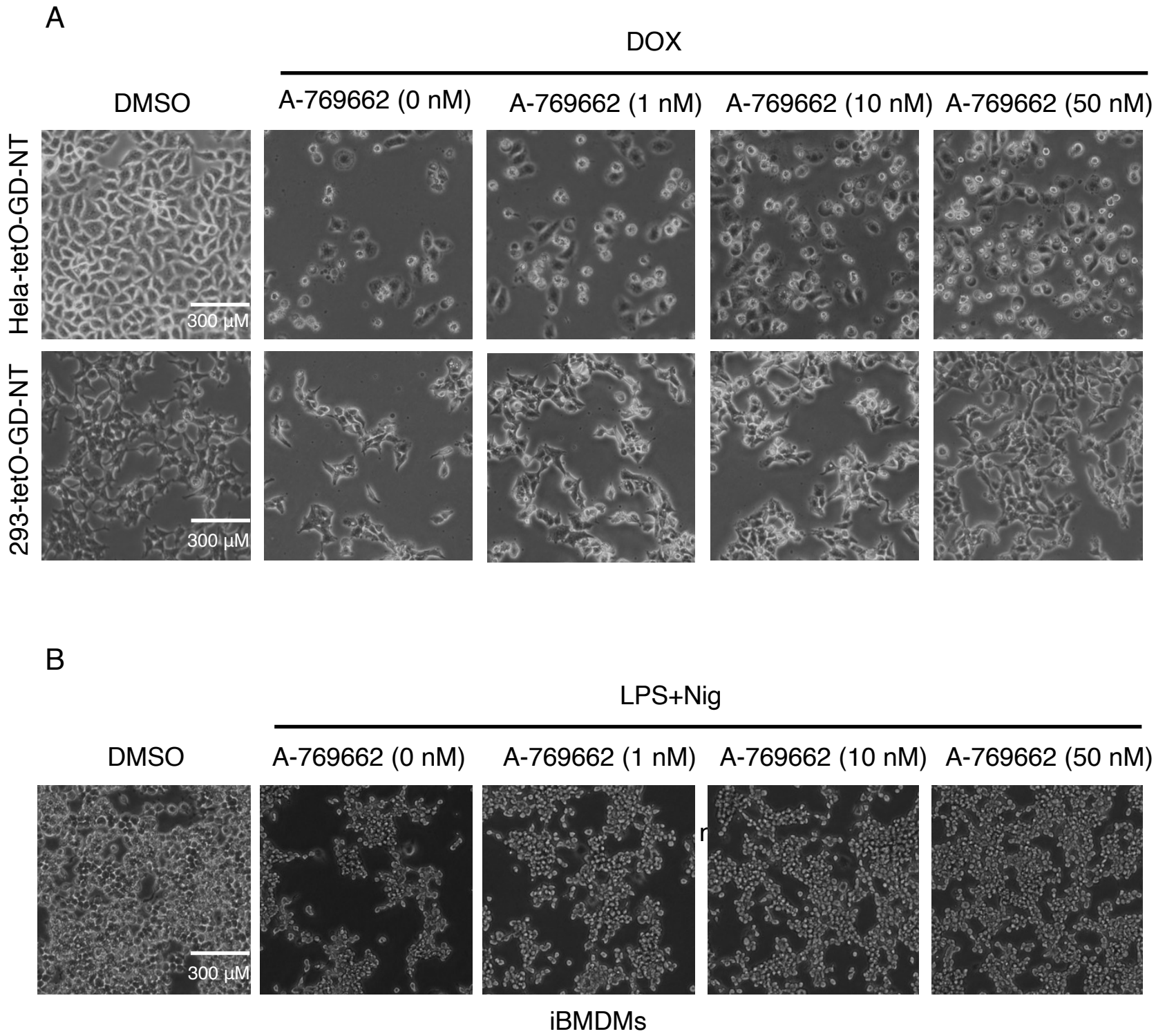

Supplementary Figure 1

A-769662 inhibits GD - NT - mediated pyroptosis in different cells. (A) HeLa-tetO-GD-NT cells were treated with Dox (2 mg/ml) to induc e pyroptosis. Different concentrations of A-769662 were added simultaneously with Dox. 18 hours later, the cells were then imaged usi ng phase-contrast microscopy. (B) iBMDM cells were !rst primed with LPS (1 mg/ml), followed by the addition of Nigericin (2 mM) to in duce pyroptosis and A!769662 at concentrations of 0 nM, 1 nM, 10 nM, and 50 nM together with LPS. After treatment with Nigericin for 6 h, proteins were collected for Western blot analysis.
